# Road safety practices among school-going children in Chennai

**DOI:** 10.1186/1865-1380-7-S1-O2

**Published:** 2014-07-25

**Authors:** Swathi Padankatti, Uma Narayanan, Jisha Susan Babu, Kuruvilla Thomas

**Affiliations:** 1 Sundaram Medical Foundation Dr Rangarajan Memorial Hospital, Shanthi Colony, Anna Nagar, Chennai, India

## Objective

To study the awareness and execution of road safety practices among school children.

## Methods

Design: Questionnaire based study

Setting & Participants: Children studying in 9th & 10th grades of city schools in Chennai, Tamilnadu, India.

## Results

Questionnaires were completed by 234 (93.6%) of 250 children who were approached. The mean age of the children was 14.3 (range 13-15 years). 70 (29%) were girls and 141 (60%) were boys; 23 (9.8%) did not fill in the personal details. 12 girls had driven a car or bike, of which 6 always, 4 sometimes and 2 never had driven without a helmet/seat-belt. 66 boys had driven a car or bike of which 32 has always, 22 sometimes and 12 had never driven without a helmet/seat-belt.

33 (47%) girls and 90 (63.8%) boys had ridden as pillion, of which only 5 boys and no girls wore a helmet. 17 (6.8%) children did not answer this question. 31 (21.9%) boys and 14 (2%) girls had had a road traffic accident (RTA); 4 boys and no girls sustained fractures. None of the children had a license. 34 (24%) boys and 17 (24%) girls used seat-belts in the passenger seat of cars. 10 boys and no girls had used mobile phones when driving. 10 (7%) boys and 11 (15%) girls admitted to disregarding traffic signals. 58 (41%) boys and 28 (40%) girls had friends who had an RTA. 22 (15%) boys and 2 (2%) girls had involved in racing while driving. None had consumed alcohol while driving.

116 (82%) boys and 49 (70%) girls rode bicycles on the road, 2 (4%) girls and 5 (4.3%) boys used helmets. 48 mothers and 115 fathers of these children used helmets while driving two wheelers: 60 mothers and 107 fathers used seat-belts while driving cars. 122 (86%) boys and 59 (84.2%) girls believed that helmets and seat-belts prevent serious injury.

## Limitations

Doing a cluster sampling of all the city schools would have yielded a more holistic picture.

## Conclusion

The non-use of seat-belts, helmets, unlicensed driving of vehicles and scant regard to traffic rules are rampant in school-going children. The law regarding this has to be enforced and punishment maximised to improve road safety practices.

